# An application of the Theory of Planned Behavior within a living organ donation education intervention for American Muslim communities: a pre–post evaluation

**DOI:** 10.1093/her/cyag030

**Published:** 2026-10-16

**Authors:** Rosie Duivenbode, Milda Saunders, Raudah Yunus, Michael T Quinn, Aasim I Padela

**Affiliations:** Department of Sociology, University of Uppsala, Engelska Parken, Thunbergsvägen 3H, 752 38 Uppsala, Sweden; Department of Medicine, University of Chicago Medicine, 5841 South Maryland Avenue, MC 6092, Chicago, IL 60637, United States; Emergency Medicine Department, Medical College of Wisconsin, 8701 Watertown Plank Rd, Milwaukee, WI 53224, United States; Department of Public Health Medicine, Faculty of Medicine, Universiti Teknologi MARA, Sungai Buloh Campus, 47000 Jalan Hospital, Selangor Darul Ehsan, Malaysia; Department of Medicine, University of Chicago Medicine, 5841 South Maryland Avenue, MC 6092, Chicago, IL 60637, United States; Emergency Medicine Department, Medical College of Wisconsin, 8701 Watertown Plank Rd, Milwaukee, WI 53224, United States

## Abstract

The disparity between supply and demand for life-saving and life-sustaining donor organs disproportionally affects ethnic and racial minorities, which is partly due to negative attitudes and beliefs regarding transplantation. One way to address these disparities is to implement targeted and tailored behavioral interventions among minority communities, such as the American Muslim population. We designed, implemented, and evaluated a religiously tailored, ethically balanced, and mosque-based educational intervention within the USA. This intervention increased participants’ biomedical and religious knowledge of living organ donation, self-perceived preparedness to make a donation-related decision, and self-reported likelihood to donate a kidney. This paper assesses whether these reported outcomes can be explained through the Theory of Planned Behavior. Our intervention positively impacted living organ donation-related behavioral, normative, and control beliefs—independent of sociodemographic factors. These changes were partly associated with changes in the intention to perform donation-related behaviors. Most significantly, a positive change in behavioral beliefs increased participant’s self-perceived likelihood to donate a kidney during their lifetime. These findings support the application of the Theory of Planned Behavior to educational interventions on organ donation in religious communities and could support those seeking to design theory-driven educational interventions for Muslim minorities in other western contexts.

## Introduction

The disparity between supply and demand for life-saving and life-sustaining donor organs persists globally. With over 100 000 individuals currently on the US national transplant waiting list, this disparity will remain a significant public health concern for the foreseeable future [1]. Ethnic and racial minorities are disproportionally affected by donor organ shortages. Two important explanations for this observation are the increased difficulty in finding appropriate matches due to biological factors and fewer donations from individuals from these minority backgrounds [2]. At the same time, the need for organs among minoritized groups is higher when compared to the non-Hispanic White population given that several diseases that are considered risk factors for organ failure—such as diabetes, obesity, and hypertension—are observed in higher rates [3–9]. One way to address this donor disparity is to implement targeted and tailored programs that facilitate informed organ donation decision-making among such groups.

The American Muslim population, currently estimated to be around 4.5 million people and projected to grow to 8.1 million by 2050, is one minority community that could benefit from educational interventions that attend to their knowledge gaps and cultural values [10, 11]. American Muslims are one of the most racially diverse religious groups, with no single racial or ethnic group forming a clear majority [12, 13]. While there are scant data on a national level on Muslim Americans, research shows that some of the most predominant ethnic/racial sub communities—namely, South Asian Americans (30%), indigenous African Americans (20%), and Arab Americans (12%)—are disproportionally affected by donor organ shortages, making organ transplantation a Muslim community health priority [12, 14]. Furthermore, a large proportion of American Muslims rate religion as important in their daily lives (between 60% and 89%) and they are known to hold negative attitudes toward organ donation [12, 13] Illustratively, while over 95% of the American population supports organ donation, the reported support for donation among American Muslims ranges from 20% to 51% [15–17]. Previous research has revealed a general lack of awareness of the importance of and need for organ donation, as well as religious knowledge gaps regarding the (im)permissibility of donating [18–21]. These factors likely contribute to the lower rates of support for organ donation among Muslim Americans.

The lower support for organ donation found among American Muslims corresponds with other diasporic Muslim communities, as well as with Muslim majorities countries across the globe. For example, a study among South Asians living in the UK found that Muslims were less likely than Hindus of Sikhs to support organ donation or to register as a donor and more likely to report religious guidance as important [20]. A study among individuals from different religious denominations in Australia found that those with stronger religious beliefs had more negative attitudes toward organ donation, particularly among Buddhist and Muslim respondents [22]. A meta-analysis assessing willingness to donate in various Muslim majority countries across the Middle East (specifically Iran, Saudi Arabia, Qatar, and Morocco) found that the pooled willingness to donate was just under 50% (range 24.5%–89.0%) [23] More recent studies support these findings, with relatively low reported willingness to register as an organ donor in, for example, the United Arab Emirates (58%), Yemen (52.5%), and Saudi Arabia (21.2%–26%) [24–27].

Informed by this academic literature, we designed, implemented, and evaluated a religiously tailored, ethically balanced, and mosque based educational intervention within the USA, entitled “Informing American Muslims about Living Donation” (I AM a LD). This overarching study was a randomized controlled cross-over trial aimed at increasing biomedical and religious knowledge of organ donation, changing beliefs related to organ donation, and improving preparedness and intention to perform donation-related behaviors. The overall results, published elsewhere, demonstrated that biomedical and religious knowledge gaps can be feasibly addressed by a religiously tailored intervention in mosque settings [28]. In addition, the intervention positively impacted participant stage of change (model), preparedness to make a donation decision, and self-reported likelihood to donate a kidney [29]. The paper presented herein assesses, through a pre–post analysis, whether the positive effects of the intervention workshop can be explained through the Theory of Planned Behavior—as we hypothesized in our study design [30]. In other words, did the educational intervention positively impact organ donation-related behavioral, normative, and control beliefs? And, if so, were these changes associated with changes in the intention to perform donation-related behaviors?

## Methods

### Theoretical foundation and supporting data

Both the design of the educational intervention and the outcome measures were informed by the Theory of Planned Behavior (TPB) [31]. The TPB asserts that having the intention to perform a behavior is the strongest predictor of its performance when the opportunity arises. In turn, an individual’s intention to perform a certain behavior will be stronger if one’s attitude (favorable or unfavorable evaluation of the behavior), subjective norm (perceived social inclination to perform the behavior), and perceived behavioral control (proxy of actual control) are more favorable. The foundations for these three domains are—in order—behavioral beliefs, normative beliefs, and control beliefs, i.e. beliefs about the outcomes of the behaviors (both positive and negative), the normative expectations of social referents (including religious referents), and the perceived presence of factors that might facilitate or impede its performance (e.g. skills and resources). For a visual overview of the TPB, please refer to Fig. 1.

**Figure 1:**
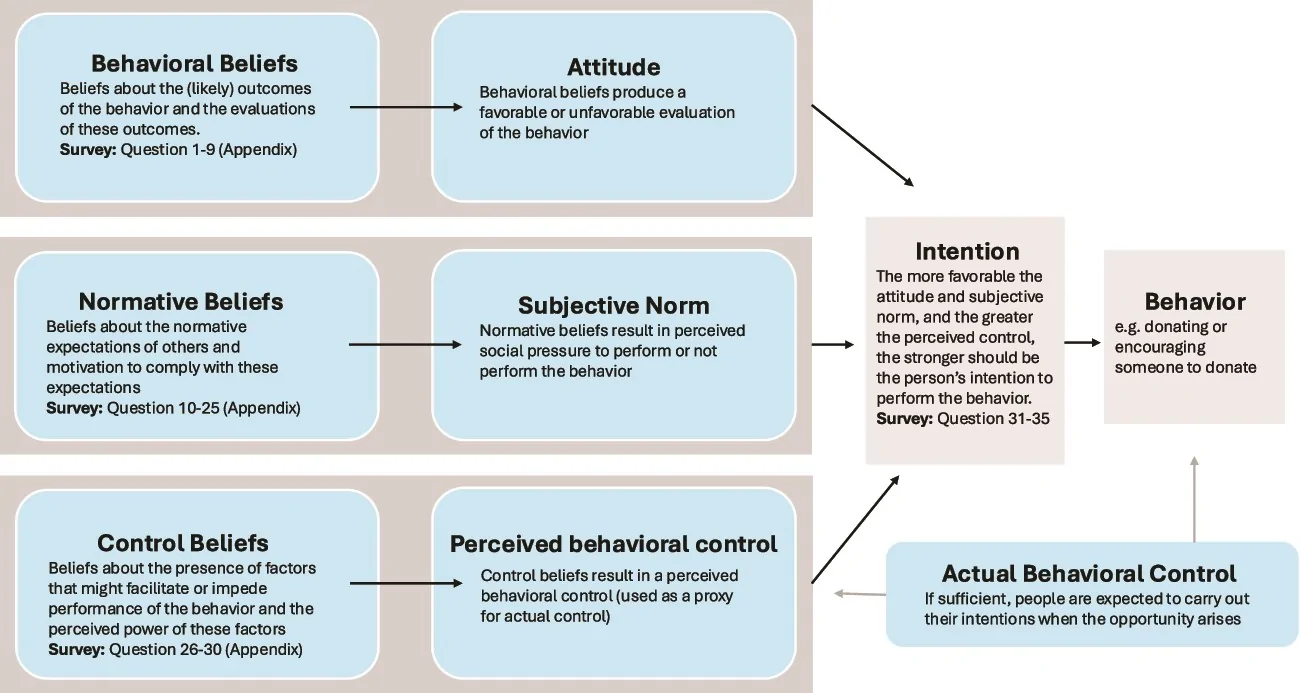
Theory of Planned Behavior constructs and survey items.

The TPB has been employed for understanding various health behaviors, including living and deceased organ donation [32–35]. Given that religion is known to impact organ donation decision-making, attempts have been made to incorporate religious beliefs into the TPB. One study, for example, created an extended 3D attitude measure covering outcome expectations (the traditional operationalization of the TPB attitude domain), religious attitudes, and affective attitudes [36]. They found that religious attitudes added unique predictive ability. The study presented in this paper has conceptualized religious beliefs within the traditional structure of the TPB. For example, religious attitudes were (re)formulated as outcome expectations such as “if I were to donate an organ, I would be giving something away that is not mine to give” (please refer to the survey items in the appendix for more examples).

Our previous formative qualitative research among Muslim Americans drew on the TPB to identify salient behavioral, normative, and control beliefs—paying special attention to religiously informed beliefs [18]. The TPB was employed both in the design of that study’s interview guide and qualitative analysis. While the interview guide had only expanded the normative domain to include religious beliefs, the analysis revealed that religiously informed ideas were not restricted to this domain [18]. We carried these findings and identified beliefs forward into the present study design. More specifically, the TPB beliefs identified in our earlier research (both those that were religiously informed and those that were not) represent hypothesized mediators for “I AM a LD” research participants’ intention to perform donation-related behaviors.

### Study design, setting, and participants

As a secondary data analysis of the “I AM a LD” randomized controlled cross-over trial, a pre–post analysis of participant data was conducted to assess whether the previously established impact of the educational intervention could be explained through the TPB. The study was conducted across four mosques in the Washington DC and Chicago metropolitan areas. These mosques were selected based on their large and diverse ethnic and racial Muslim populations and prior agreement to participate in the study by their boards of directors. All four mosques facilitated the five daily congregational prayers, as well as Friday prayers (between two and four sessions in each mosque). None had hosted education programs on organ donation or transplantation prior to our intervention.

Study participants were Muslim Americans attending these four mosques: they were recruited *via* staffed tables at Friday prayers and other mosque events. This was supplemented by word-of-mouth recruitment and announcements in mosque newsletters, social media, and listservs. Interested mosque attendees left their contact details and received a follow-up call by a research assistant to ensure eligibility and obtain oral informed consent. To be included, participants had to identify as Muslim, be 18 years or older, and be proficient in English. They were excluded if they had a positive donor history—defined as having personally donated or received an organ or having a close family member (parent, sibling, child) who had done so—or were unable to attend the workshop at the designated days. Participants received a total of $60 in gift cards for attending two half-day workshops (intervention and control) as remuneration. The order of the intervention (organ donation) and control (end-of-life care) workshops was randomized at the mosque level. Ethical approval and oversight were provided by the Institutional Review Board of the Biological Sciences Division of the University of Chicago.

### Intervention design

The intervention workshop lasted ~5 h on a weekend morning. The program included both didactic teaching on the biomedical (by a living donor advocate physician), procedural (by an organ donation professional), and religious (by an Islamic bioethicist) dimensions of organ donation and a peer-led group discussion (see Table 1 for an example schedule). All participants received a physical course book with the program, presentation slides, and a *fatwa* (religious opinion) on organ donation and transplantation issued by the Fiqh Council of North America [37]. The educational content was ethically balanced—discussing the biomedical pros and cons, and the religious arguments in favor of and against organ transplantation—and thus aimed to enhance informed decision-making among individual participants. The biomedical, procedural, and religious didactic teaching was delivered by the same experts at each site. Group discussions were led by trained Muslim peer educators from each locale. During a 1-day face-to-face intensive and an online webinar refresher prior to the workshops, the peer educators reviewed the educational material and were taught to facilitate free-flowing conversations on the pros and cons of organ donation with respect to oneself, one’s family, and larger society. Both the didactic and discussion sessions were designed to address the TPB beliefs previously identified in our qualitative study and aimed to facilitate central route processing [38]. All educational and training materials can be found on the project’s website [39].

**Table 1:** Example workshop schedule

8.30–8.45	Registration, breakfast
8.45–9.05	Welcome, Survey explanation
9.05–9.30	Pre-survey assessment
9.30–10.10	Biomedical aspects of living organ donation
10.10–10.40	Organ transplant in the USA
10.40–10.55	Coffee and tea break
10.55–11.50	Islamic perspectives on living organ donation
11.50–12.45	Facilitated group discussion
12.45–13.00	Prayer break
13.00–13.45	Closing, lunch and post-survey assessment

A table providing an example overview of the workshop schedule, with times in the left column and a brief description of the activities in the right column.

### Outcome measures

The primary survey outcomes measured change in the three TPB domains and change in intention to perform several donation-related behaviors (see Fig. 1 for a reference to the survey items per domain; survey items can be found in the appendix). The TPB domain scales were based on our prior focus group data [18]. These data revealed several behavioral, control, and normative beliefs—many of them directly or indirectly relating to religious considerations—which were rewritten as survey questions. Items from each belief domain composed three additive scales (nine behavioral items, seven control items, and eight normative items) and were revised for clarity through expert panel review. For each normative item, a “motivation to comply” item was included that assessed the importance of the social referents’ opinion to the participants. Participants were asked to rate the statements (e.g. “If I were to donate an organ, I would be performing a good deed”) on a 4-point agreement scale (from completely disagree to completely agree).

We developed five items that evaluated the intention to perform several donation-related behaviors. The first item was modelled on the Transtheoretical Model (stages of change) and read: “Thinking about where you are today, which of the following best describes your thoughts on living organ donation?” (ranging from “I have never thought about it” to “I have thought about it a lot, and I am ready to make a decision if needed”) [40]. The other four items assessed behavioral likelihood by asking “Please indicate how likely you are to do each of the following actions in the future: i) donate your kidney, ii) encourage a loved one with end-stage renal disease to seek out a donor, iii) encourage a co-worker with end-stage renal disease to seek out a donor, iv) encourage someone at your mosque with end-stage renal disease to seek out a donor?” Each item was rated on a 4-point Likert-type scale (ranging from extremely likely to extremely unlikely).

### Independent measures

The independent measures consisted of (i) sociodemographic characteristics, (ii) religious characteristics, and (iii) previous organ donation experience (i.e. knowing someone who had either donated or received an organ who was not a direct family member, see exclusion criteria). Sociodemographic characteristics included those known to be associated with organ donation attitudes (age, sex, race/ethnicity, health), as well as acculturation proxies (country of origin, primary language). Given that the intervention was specifically designed for a religious community, religiosity was measured comprehensively through three different scales. First, the Duke University Religion Index (DUREL) was modified for Muslim respondents by adjusting frequency categories of congregational religious services and by replacing certain words for more commonly used vocabulary among this group, e.g. God with Allah [41]. Second, Islamic religiosity was measured through two subscales of the Psychological Measure of Islamic Religiousness (PMIR), namely, positive and negative religious coping [42]. The seven-item PMIR-Positive Religious Coping and Identification subscale measures the extent to which Muslims use religious coping methods (e.g. reading Qur’an, seeking forgiveness, cultivating reliance upon God) to deal with life stressors. The three-item PMIR-Punishing Allah Reappraisal subscale assesses whether people interpret events in their life as punishment from God (negative religious coping). This subscale is known to correlate negatively with organ donation attitude among Muslims [16]. Both scales were slightly adapted by changing the answer options from the original Likert-type scale ranging from 1 “I do not do this at all” to 4 “I do this a lot” to a 4-point Likert-type agreement scale ranging from 1 “completely disagree” to 4 “completely agree.” Previous organ donation knowledge (other than the exclusion criteria) was assessed by asking (i) whether participants were designated donors and (ii) if they had a relative or close friend who needed an organ transplant, has received a donor organ, or has donated an organ. Lastly, having demonstrated that the intervention resulted in a change in biomedical and religious organ donation knowledge among participants elsewhere, knowledge change was employed as an independent measure for the purposes of this sub-study [28]. Religious knowledge was measured using our previously validated Islamic Knowledge of Living Organ Donation (IK-LOD) scale, and biomedical knowledge was assessed through the living donation subscale of the Rotterdam Renal Replacement Knowledge Test (R3KT) [43, 44].

### Data sources, collection, and management

Data were collected *via* two anonymous but linked paper-based surveys (pre and post) completed by workshop participants during the allocated workshop time (see Table 1). To ensure accuracy, double-data entry was performed by research assistants into a REDCap database with discrepancies resolved by recourse to original paper surveys [45].

### Analytical approach and data transformation

The analyses consisted of two steps. First, a pooled single group analysis was conducted in which paired *t*-tests were used to assess whether pre–post changes in each of the TPB domains were significant, followed by bi- and multivariate regressions to assess whether any of the independent measures were predictive of this change. Second, the Spearman’s Rho correlation coefficient (*r*_s_) was calculated to evaluate whether there was an association between change in each TPB domain and change in the five intention items, i.e. did a change in TPB beliefs result in a change in the proxies of organ donation intention?

Change in outcome measures, i.e. each of the three TPB domains and the five intention items, was calculated by subtracting pre-survey from post-survey scale or item scores. As the TPB scales were created *de novo*, we first assessed the quality of the scales and adjusted them accordingly. This was done by evaluating the relationship between items and the overall scale (Pearson, item–test, and item–rest correlations) and performing an internal consistency reliability test using Cronbach’s α statistics before conducting further analyses. The behavioral beliefs and normative beliefs scales had Cronbach’s α statistics of 0.795 and 0.883, respectively, and were kept as initially designed. The control beliefs scale had a Cronbach’s α of 0.665 with two items standing out for review (low item–test and item–rest correlations): “If I were to consider living donation it is important for me to know my organ i) does not go to someone with anti-Muslim or Islamophobic attitudes, and ii) goes to someone who is Muslim.” Although these items were based on concerns identified in our previous qualitative research, upon further reflection they did not accurately capture “perceived control” as defined by the TPB. Rather they represented broader relational concerns and preferences of a marginalized minority. Here, conceptual and statistical justifications supported their exclusion. Excluding these two items resulted in a five-item scale with a Cronbach’s α statistic of 0.863.

The following sociodemographic scales were collapsed due to low numbers: ethnic background into South Asian and other, and primary language spoken at home into English, Urdu, and other. In addition, an acculturation measure was developed by combining “country of origin” and “primary language spoken at home” into a summed score to facilitate analysis.

## Results

### Analytic sample and data transformation

Study staff registered a total of 833 interested individuals across the four mosque sites (see Fig. 2 for the participant flow diagram). During the follow-up phone calls, more than half were either ineligible, unable to be contacted, or declined to participate. There were 337 study participants enrolled with a 67% participation rate. Out of this group, 158 attended the first workshop session in the series, i.e. either the control or intervention workshop, resulting in a 47% attendance rate. The analytical sample for this manuscript includes the 136 participants who attended the organ donation workshop and completed the surveys (assessment response rate 100%). Full participant characteristic data can be found in Table 2. Of note, the final analytic sample had a uniform distribution regarding biological sex and age, and was predominantly of south Asian ethnicity (over 80%), and the majority was born outside of the USA (over 75%). Yet, of those born abroad, a majority, just over 67%, had lived in the country for >20 years.

**Figure 2:**
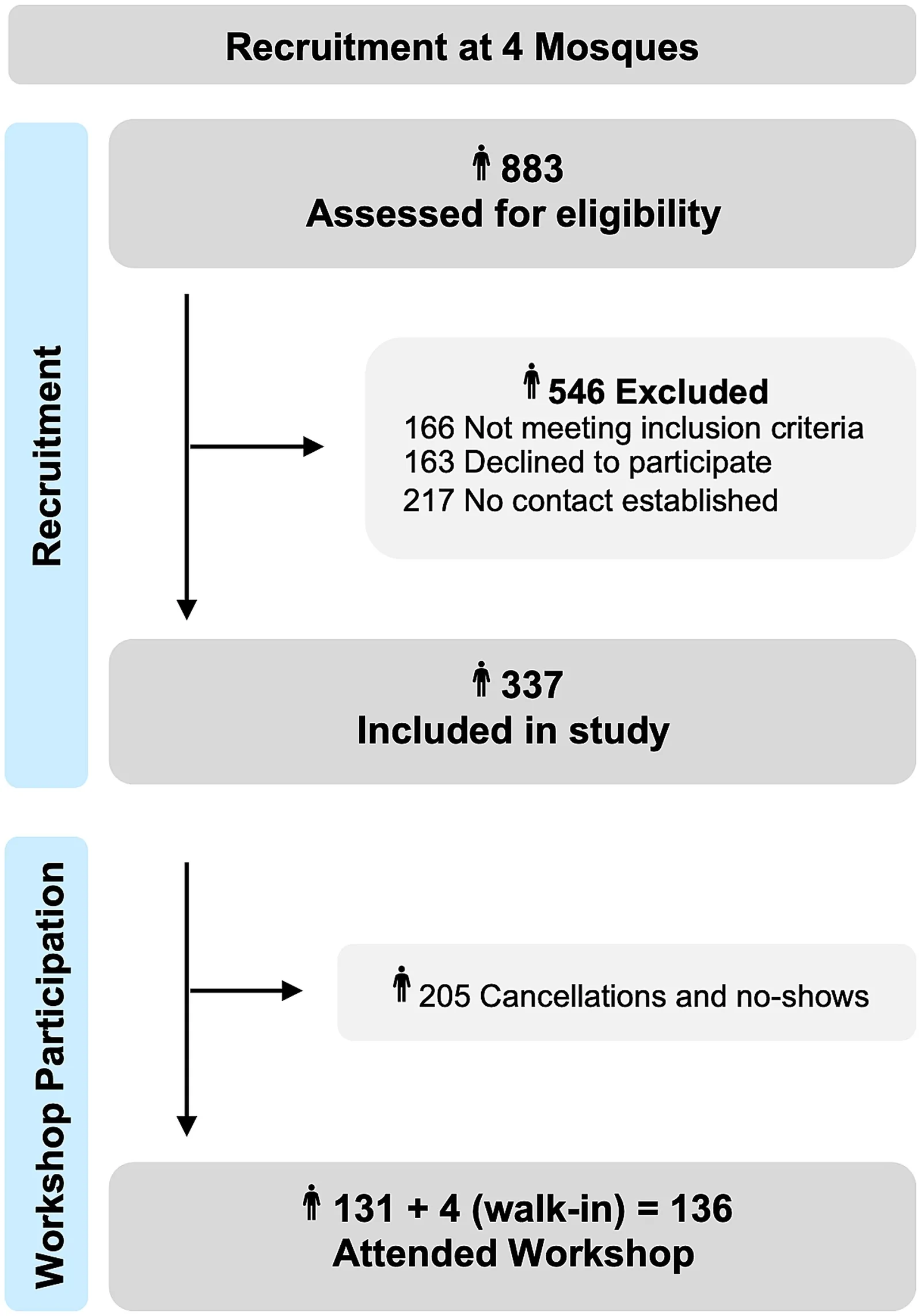
Participant flow diagram.

**Table 2:** Participant characteristics

Characteristic	No. (%)
**I. Socio-demographic information (*n* = 136)**	
Sex (*n* = 131)	
Male	59 (45.0)
Female	72 (55.0)
Ethnicity (*n* = 130)	
South Asian	109 (83.9)
Other	21 (16.2)
Age (*n* = 130)	
18–30 years old	27 (20.8)
31–40 years old	23 (17.7)
41–50 years old	28 (21.5)
51–60 years old	19 (14.6)
61–70 years old	19 (14.6)
71–85 years old	14 (10.8)
Country of origin (*n* = 130)	
USA	32 (24.6)
Other	98 (75.4)
Residency in the USA (*n* = 101)	
Less than 5 years	9 (8.9)
6–10 years	5 (5.0)
11–15 years	7 (6.9)
16–20 years	12 (11.9)
More than 20 years	68 (67.3)
Primary language (*n* = 130)	
English	59 (45.4)
Urdu	52 (40.0)
Other	19 (14.6)
**II. Religiosity**	
Islamic affiliation (*n* = 131)	
Sunni	128 (97.7)
Psychological Measure of Islamic Religiousness (PMIR)^a^	
Positive religious coping, mean (range) (*n* = 129)	26.4 (7, 28)
Negative religious coping, mean (range) (*n* = 125)	6.4 (3, 12)
Duke University Religion Index (DUREL), mean (range) (*n* = 127)^a^	23.2 (6, 27)
**III. Organ donation experience**	
Designated donor (*n* = 132)	
Yes	22 (16.7)
Prior donation-related experience (*n* = 131)	
Yes	39 (29.8)
**IV. Health (short form 1) (*n* = 132)**	
Excellent	33 (25.0)
Very good	39 (29.6)
Good	40 (30.3)
Fair	20 (15.2)

The table contains pooled participant characteristics, namely, sociodemographic characteristics, religiosity, organ donation experience, and health, with their numerical frequency.

a The PMIR and DUREL measures were slightly adapted (see main text for details).

### Part I: TPB domains

A complete case analysis was performed. Paired *t*-tests were used to assess whether pre–post changes in each of the TPB domains were significant. As shown in Table 3, a statistically significant change in behavioral (Δ0.88, *P* = .007), normative (Δ1.55, *P* = .0002), and control (Δ1.26, *P* < .0001) beliefs was observed, following the educational workshop. Bivariable analyses identified several variables associated with change in organ donation beliefs. For behavioral beliefs, an increase in either religious (*P* = .033) or biomedical (*P* = .026) knowledge was significantly associated with a positive change. With respect to normative beliefs, PMIR-negative religious coping (*P* = .028) and acculturation (*P* = .006) were associated with positive change in beliefs. For control beliefs, biomedical knowledge (*P* = .022) and ethnicity (*P* = .014) were predictive of positive change. Due to these significant correlations, these variables were then included in a multivariate ordinary least squares (OLD) regression modeling (see Table 4), along with others that had *P*-values of <.1 to ensure that we captured all potential predictors. Upon multivariate modeling, only one independent predictor for change in normative beliefs was found, namely, low acculturation (*P* = .023).

**Table 3:** Change in attitudes toward organ transplantation according to TPB domain

*TPB domain scale*		Baseline	Post	Δ	*P*-value
	*n*	Mean (SD)			
Behavioral Beliefs	131	28.26 (4.82)	29.13 (4.49)	0.88	.007
Normative Beliefs ^*^ Motivation to Comply (MTC)	123	17.46 (5.15)	19.0 (5.60)	1.55	.0002
Control Beliefs	126	16.18 (3.09)	17.44 (2.65)	1.26	<.0001

Table displays the baseline and post-intervention scores for each of the three TPB domains (behavioral, normative, and control beliefs), as well as a *P*-value to determine the significance of the change in attitudes.

**Table 4:** OLD regression modeling of predictors on changed TPB domain beliefs

*Model*	n	Coefficient (95% CI)	*P*-value
I. Behavioral Beliefs	114		
Δ IK-LOD (Islamic knowledge)		0.24 (−0.06, 0.53)	.118
Δ R3KT (biomedical knowledge)		0.19 (−0.09, 0.48)	.177
II. Normative Beliefs ^*^ MTC	110		
PMIR Negative		0.22 (−0.05, 0.49)	.109
PMIR Positive		0.08 (−0.32, 0.48)	.685
Acculturation			
Low		2.40 (0.344, 0.46)	.023^**^
Medium		0.93 (−1.30, 3.16)	.411
High		REF	REF
III. Control Beliefs	107		
Δ R3KT (biomedical knowledge)		0.18 (−0.03, 0.39)	.090^*^
PMIR Positive		−0.09 (−0.36, 0.17)	.495
Health Short Form 1		0.42 (−0.09, 0.92)	.105
Ethnicity (South Asian)		−1.23 (−2.69, 0.23)	.10

^*^ *P* < .1.

^**^ *P* < .05.

The table displays the outcome of regression modeling with predictors for the three TPB domains (behavioral, normative, and control beliefs) with coefficients and *P*-values.

### Part II: Behavioral intention

The skewness/kurtosis test for normality found that scores for change in intention across items 3, 4, and 5 were skewed (see footnotes Table 5 for each item). Spearman’s test was used to examine the relationship between change in TPB beliefs and change in intent (see Table 5). A change in behavioral beliefs correlated with an increased likelihood to donate one’s own kidney (*P* = .0197, *r*_s_ 0.211). In addition, a change in control beliefs was associated with increased likelihood to encourage a loved one with end-stage renal disease to seek out a living donor (*P* = .018, *r*_s_ 0.216). No other statistically significant associations between change in TPB domain and intention measures were found.

**Table 5:** Association between change in TBP beliefs and change in intention to perform donation-related behaviors

*TPB domain scale*		Stages of change^a^	Likelihood—self^b^	Likelihood—loved one^c^	Likelihood— coworker^d^	Likelihood—mosque member^e^
Behavioral Beliefs	*n*	122	122	120	122	121
	*P*-value	.702	.0197^**^	.249	.839	.365
	*r*_s_		0.211			
Normative Beliefs ^*^ MTC	*n*	117	117	117	117	117
	*P*-value	.820	.733	.131	.578	.483
	*r*_s_					
Control Beliefs	*n*	120	121	119	121	120
	*P*-value	.125	.176	.018^**^	.195	0.287
	*r*_s_			0.216		

This table shows the association between change in each of the three TPB belief domains and change in five different intention measures.

a Thinking about where you are today, which of the following best describes your thoughts on living organ donation?

b How likely are you to donate your kidney?

c How likely are you to encourage a loved one with end-stage renal disease (ESRD) to seek out a donor?

d How likely are you to encourage a co-worker with end-stage renal disease (ESRD) to seek out a donor?

e How likely are you to encourage someone at your mosque with end-stage renal disease (ESRD) to seek out a donor? ^**^statistically significant.

## Discussion

To conclude, this paper assessed whether the previously published positive outcomes of the “I AM A LD” educational intervention, namely, change in participant stage of change (model) regarding organ donation, preparedness to make a donation-related decision, and self-reported likelihood to donate a kidney, could be explained through changes in behavioral, normative, and control beliefs as asserted by the TPB [30]. The results presented here show that the intervention did indeed positively impact organ donation-related behavioral, normative, and control beliefs among program participants, as a statistically significant change in all three domains was observed. At the same time, these changes were indeed, at least partly, associated with change in participants’ intention to perform donation-related behaviors. Namely, (i) a positive change in behavioral beliefs (i.e. outcome expectations) was associated with an increase in research participants’ self-reported likelihood to donate a kidney, and (ii) a positive change in perceived behavioral control was associated with an increase in reported likelihood to encourage a loved one to seek out a donor. However, no other associations between the belief domains and intention measures were found. These findings—despite having only partial explanatory power—are consistent with the TPB model of behavior change, supporting the theory’s application to educational interventions related to donation within a religious community.

The partial explanatory power demonstrated in this paper is in line with prior studies that have attempted to “match” empirical data with the constructs and pattern of TPB [36, 46]. In a systematic review of 17 studies on TBP application on organ donation behaviors (involving 10 118 subjects across eight countries), only 3 studies had results that could adequately explain the relationships between ‘all’ the constructs within TPB (i.e. where attitude, subjective norms, and perceived behavioral control were associated with, or predicted, intention to perform organ donation–related behaviors). The rest only partially explained the model [46]. Among these, some studies found associations between one or two—not all—of the belief domains with intention across different ethnic groups [34, 47], while others suggested different pathways to explain the links between TPB constructs [36, 48, 49]. Others have added new variables—such as affective attitudes, self-identity, moral norms, and anticipated regret—into the original model to form what is known as extended or augmented forms of the TPB framework because they found that existing constructs are not sufficient to predict organ donation-related intentions [36, 50, 51].

These mixed findings challenge the standard TPB framework, as empirical realities do not fully match with the theory. Yet, the incongruences can be attributed to several factors. The first is cultural, as different cultures may privilege one TPB construct over the other in forming intentions to donate, due to the (distinct) meaning and implication they attach to a particular behavior [47]. The second factor is the issue of measurement heterogeneity. For instance, it has been argued that subjective norms are said to comprise two types of norms—descriptive (i.e. what most others do) and injunctive (what most others approve or disapprove of)—but different studies might incorporate one over the other [36, 52]. This renders comparison across studies difficult and might suggest some measurements of subjective norm are incomplete.

Given that in our study the measurement of normative beliefs only addressed injunctive norms (the approval/disapproval of important referents with regards to organ donation) and not descriptive norms (whether those people engage in organ donation too), the lack of association between normative beliefs and intentions could potentially be explained by an incomplete measurement of subjective norm. In other words, it is possible that even though our participants perceive that people who matter to them approve of organ donation, they see few real-life examples among their Muslim community members who have engaged in organ donation. This means that the positive change in normative beliefs we measured may not have led to a change in intention due to a lack of change in descriptive norms.

A third factor that might explain the incongruence between TPB and empirical data is that TPB constructs can vary in their importance across behaviors and situations. Icek Ajzen, who developed the TPB together with Martin Fishbein, maintained that “in some applications it may be found that only attitudes have a significant impact on intentions, in others that attitudes and PBC are sufficient to account for intentions, and in still others that all three predictors make independent contributions” [31]. Relating this to our findings, we can say that behavioral beliefs (or attitudes) may be a more important determinant for a Muslim’s intent to donate an organ, compared to their perceived ease or difficulty to donate and perceived social pressure. This suggests that the biggest barrier to living donation among Muslim Americans might be behavior beliefs grounded in religious notions of health and donation (see survey items in appendix), most notably the assumptions that all types of organ donation are categorically prohibited in Islam. Based on these findings, we suggest that nuancing this component (i.e. behavioral beliefs or attitude) in educational interventions could exert a more meaningful impact on Muslim Americans’ intention to donate.

It should be noted that our intervention was not to directly promote organ donation but rather to support informed decision-making (grounded in a balanced education regarding religious pros and cons and biomedical risks and benefits). We prioritized taking seriously the religious, cultural, and medical concerns the Muslim population might have regarding organ donation—rather than aiming to steer them toward donation through one-sided information to promote donation. While we did see a positive change, we thus allowed for donation-related intent to vary during the intervention, and this could be an additional reason why our data might not fully justify the TPB model.

While the results of this study do not completely conform to the TPB scheme, our findings nonetheless contribute to the growing evidence for TPB to be an effective theoretical framework to base behavior change interventions on. While we acknowledge this is not the first empirical study on organ donation that attempts to justify the TPB, the referenced systematic review by Latifi *et al*. [45] shows that across all the studies gathered, only one adopted an experimental design; most others were cross-sectional. Our study therefore contributes to the availability of more robust data derived from a rigorous randomized controlled trial design that can be reliably used to demonstrate causal relations within a theoretical framework. In addition, we focused on a faith minority that is relatively understudied in the USA and provide insight into how TPB might work to explain and intervene upon organ donation intentions among Muslim minorities. Our findings could thus provide insight for those who seek to design theory-driven educational interventions for Muslim minorities in other western contexts, as well as Muslims across the globe overall.

At the same time, there are several potential limitations to the study’s generalizability. First, the generalizability of the results is constraint by the study population. Although the sample was designed to reflect American Muslim communities in the Washington, DC, and Chicagoland regions, the findings may not extend to other Muslim diaspora populations within the country and beyond. Differences in migration histories, socioeconomic and political contexts, levels of religiosity, healthcare systems, and acculturation processes may influence the effectiveness of the intervention across populations. Second, the intervention workshop was comprised of both its educational content and the people who delivered it. While the curriculum can be replicated through the dissemination guide (see project website), it is not possible to disentangle the effect of the educators and peer facilitators from the content itself. Consequently, the change in TPB beliefs and intentions might vary depending on who delivers the program.

In addition, an important limitation of the analysis is that it does not provide insight into which specific beliefs drove change in intentions. By grouping the behavioral, control, and normative beliefs as scales rather than analyzing them as individual items, no conclusions can be drawn regarding whether specific beliefs are responsible for the change and, if so, which ones. That said, the intervention was designed to facilitate informed decision-making, recognizing that meaningful change in intention, beliefs, and behavior is a complex and multi-dimensional process.

Based on these limitations, we have three main recommendations for future research: (i) to include a more diverse sample in any interventional studies and (ii) to employ a qualitative approach to understand in greater depth Muslims’ beliefs and attitudes toward organ donation and the relationships between TPB constructs. Finally, (iii) we suggest including extra variables that have been empirically tested and highlighted as important in predicting intentions to perform organ donation-related behaviors, such as moral norms and distrust in the healthcare system [50, 53, 54].

## Supplementary Material

Appendix_-_Survey_items_cyag030

## Data Availability

The data underlying this article will be shared on reasonable request to the corresponding author.

## References

[1] Health Resources and Services Administration, organdonor.gov , (n.d.). https://www.organdonor.gov (accessed September 28, 2025).

[2] Health Resources and Services Administration, Organ Procurement and Transplantation Network: National Data , (n.d.). https://optn.transplant.hrsa.gov/data/view-data-reports/national-data/ (accessed September 28, 2022).

[3] Santos-Longhurst A . Type 2 Diabetes Statistics and Facts. Healthline, 2014. https://www.healthline.com/health/type-2-diabetes/statistics (accessed September 28, 2022).

[4] Jaber LA, Brown MB, Hammad A et al. Epidemiology of diabetes among Arab Americans. Diabetes Care 2003;26:308–13. 10.2337/diacare.26.2.308 12547854

[5] Dallo FJ, Borrell LN. Self-reported diabetes and hypertension among Arab Americans in the United States. Ethn Dis 2006;16:699. 16937607

[6] Mohanty SA, Woolhandler S, Himmelstein DU et al. Diabetes and cardiovascular disease among Asian Indians in the United States. J Gen Intern Med 2005;20:474–8. 10.1111/j.1525-1497.2005.40294.x PMC1490101 15963176

[7] Jamil H, Fakhouri M, Dallo F et al. Self-reported heart disease among Arab and Chaldean American women residing in Southeast Michigan. Ethn Dis 2008;18:19. 18447094

[8] Jaber LA, Slaughter RL, Grunberger G. Diabetes and related metabolic risk factors among Arab Americans. Ann Pharmacother 1995;29:573–6. 10.1177/106002809502900603 7663027

[9] Burt VL, Whelton P, Roccella EJ et al. Prevalence of hypertension in the US adult population: results from the third National Health and nutrition examination survey, 1988-1991. Hypertension 1995;25:305–13. 10.1161/01.HYP.25.3.305 7875754

[10] Muslim Population by State 2026 , (n.d.). https://worldpopulationreview.com/state-rankings/muslim-population-by-state (accessed July 19, 2026).

[11] A new estimate of U.S. Muslim population | Pew Research Center , (n.d.). https://www.pewresearch.org/short-reads/2018/01/03/new-estimates-show-u-s-muslim-population-continues-to-grow/ (accessed July 19, 2026).

[12] American Muslim Poll 2025: Demographics of U.S. Muslims - Institute for Social Policy and Understanding , (n.d.). https://ispu.org/poll/american-muslim-poll-2025-full-report-2/ (accessed July 19, 2026).

[13] How US Muslims compare with other Americans on religion, age, education, race | Pew Research Center , (n.d.). https://www.pewresearch.org/short-reads/2025/06/18/how-us-muslims-compare-with-other-americans-religiously-and-demographically/ (accessed July 19, 2026).

[14] Pew Research Center, U.S . Muslims Concerned about their Place in Society, but Continue to Believe in the American Dream. Washington D.C.: Pew Research Center, 2017.

[15] Padela AI, Rasheed S, Warren GJW et al. Factors associated with positive attitudes toward organ donation in Arab Americans. Clin Transpl 2011;25:800–8. 10.1111/j.1399-0012.2010.01382.x 21175847

[16] Padela AI, Zaganjor H. Relationships between Islamic religiosity and attitude toward deceased organ donation among American Muslims: a pilot study. Transplantation 2014;97:1292–9. 10.1097/01.TP.0000441874.43007.81 24646775

[17] Hafzalah M, Azzam R, Testa G et al. Improving the potential for organ donation in an inner city Muslim American community: the impact of a religious educational intervention. Clin Transpl 2014;28:192–7. 10.1111/ctr.12296 24372808

[18] Duivenbode R, Hall S, Padela AI. A mosque-based qualitative study on American Muslim women’s organ donation beliefs. Prog Transplant 2020;30:56–62. 10.1177/1526924819893933 31896303

[19] Gauher ST, Khehar R, Rajput G et al. The factors that influence attitudes toward organ donation for transplantation among UK university students of Indian and Pakistani descent. Clin Transpl 2013;27:359–67. 10.1111/ctr.12096 23406516

[20] Karim A, Jandu S, Sharif A. A survey of south Asian attitudes to organ donation in the United Kingdom. Clin Transpl 2013;27:757–63. 10.1111/ctr.12189 23909569

[21] Sharif A, Jawad H, Nightingale P et al. A quantitative survey of western Muslim attitudes to solid organ donation. Transplantation 2011;92:1108–14. 10.1097/TP.0b013e318231ea17 21956200

[22] Wakefield CE, Reid J, Homewood J. Religious and ethnic influences on willingness to donate organs and donor behavior: an Australian perspective. Prog Transplant 2011;21:161–8. 10.1177/152692481102100213 21736247

[23] Mekkodathil A, El-Menyar A, Sathian B et al. Knowledge and willingness for organ donation in the middle eastern region: a meta-analysis. J Relig Health 2020;59:1810–23. 10.1007/s10943-019-00883-x PMC7359145 31309441

[24] Alhasan K, Aljamaan F, Ajlan A et al. Awareness, attitudes, and willingness: a cross-sectional study of organ donation in Saudi Arabia. Healthcare 2023;11:3126–42. 10.3390/healthcare11243126 PMC10742515 38132016

[25] Abdu E, Alshobi E, Mahjari A et al. Modern General Hospital, Adults’ Knowledge, Attitudes and Willingness for Organ Donation in the Yemen: A Cross-Sectional Exploration 2025. 10.2139/ssrn.5371451.

[26] Al-Bsheish M, Jarrar M, Samarkandi L et al. Attitude and personal characteristics as predictors for registration to organ donation in Saudi Arabia: a binary logistic regression model. J Med Access 2026;10:1–10. 10.1177/27550834251411661 PMC12833189 41602455

[27] Seddiq M, Uddin RH, Mohammed M et al. Knowledge, attitudes, and willingness regarding organ donation in a Muslim country: a report from the United Arab Emirates. Saudi J Kidney Dis Transpl 2023;34:482–94. 10.4103/SJKDT.SJKDT_464_20 38725198

[28] Padela AI, Duivenbode R, Quinn M et al. Informing American Muslims about living donation through tailored health education: a randomized controlled crossover trial evaluating increase in biomedical and religious knowledge. Am J Transplant 2021;21:1227–37. 10.1111/ajt.16242 32772460

[29] Padela AI, Duivenbode R, Saunders MR et al. The impact of religiously tailored and ethically balanced education on intention for living organ donation among Muslim Americans. Clin Transpl 2020;34:e14111. 10.1111/ctr.14111 33063912

[30] Padela AI, Duivenbode R, Quinn M et al. Study protocol for ‘informing American Muslims about organ donation (I AM a LD). Soc Sci Protoc 2019;2:1–17. 10.7565/ssp.2019.2654

[31] Ajzen I . The theory of planned behaviour. Organ Behav Hum Decis Process 1991;50:179–211. View At.

[32] Browne C, Desmond DM. Intention to consent to living organ donation: an exploratory study, psychol. Health Med 2008;13:605–9. 10.1080/13548500701842958 18942014

[33] Siegel JT, Alvaro EM, Lac A et al. Intentions of becoming a living organ donor among Hispanics: a theory-based approach exploring differences between living and nonliving organ donation. J Health Commun 2008;13:80–99. 10.1080/10810730701807142 18307137

[34] Bresnahan M, Lee SY, Smith SW et al. A theory of planned behavior study of college students’ intention to register as organ donors in Japan, Korea, and the United States. Health Commun 2007;21:201–11. 10.1080/10410230701307436 17567252

[35] Radecki CM, Jaccard J. Psychological aspects of organ donation: a critical review and synthesis of individual and next-of-kin donation decisions. Health Psychol 1997;16:183–95. 10.1037/0278-6133.16.2.183 9269891

[46] Rocheleau CA . Organ donation intentions and behaviors: application and extension of the theory of planned behavior. J Appl Soc Psychol 2013;43:201–13. 10.1111/j.1559-1816.2012.00998.x

[36] Auda J, Badawi J, Padela A. On Organ Donation and Transplantation, Fiqh Council of North America. Plainfield USA, 2021. https://fiqhcouncil.org/on-organ-donation-and-transplantation/ (accessed September 28, 2022).

[37] Cacioppo JT, Petty RE, Kao CF et al. Central and peripheral routes to persuasion: an individual difference perspective. J Pers Soc Psychol 1986;51:1032–43. 10.1037/0022-3514.51.5.1032

[38] Initiative on Islam and Medicine, Informing American Muslims about Living Donation [I AM a LD] , (n.d.). https://medicineandislam.org/informing-american-muslims-about-living-organ-donation/ (accessed September 28, 2025).

[39] Grimley D, Prochaska JO, Velicer WF et al. The transtheoretical model of change. In: Brinthaupt TM, Lipka RP, editors. Changing the self: Philosophies, Techniques, and Experiences, 1994;201–27.

[40] Koenig HG, Büssing A. The Duke University religion index (DUREL): a five-item measure for use in epidemiological studies. Religions (Basel) 2010;1:78–85. 10.3390/rel1010078

[41] Abu Raiya H, Pargament KI, Mahoney A et al. A psychological measure of Islamic religiousness: development and evidence for reliability and validity. Int J Psychol Relig 2008;18:291–315. 10.1080/10508610802229270

[42] Ismail SY, Timmerman L, Timman R et al. A psychometric analysis of the Rotterdam Renal Replacement Knowledge-Test (R3K-T) using item response theory. Transpl Int 2013;26:1164–72. 10.1111/tri.12188 24118241

[43] Padela AI, Duivenbode R, Quinn M. The development and validation of the Islamic knowledge of living organ donation knowledge scale for measuring organ donation knowledge among Muslim communities. Prog Transplant 2020;30:315–21. 10.1177/1526924820958119 32912075

[44] Harris PA, Taylor R, Minor BL et al. The REDCap consortium: building an international community of software platform partners. J Biomed Inform 2019;95:103208. 10.1016/j.jbi.2019.103208 PMC7254481 31078660

[45] Latifi M, Pauli J, Dehghani S et al. Application of theory of planned behavior on organ donation behavior a systematic review. Saudi J Kidney Dis Transpl 2021;32:1201–13. 10.4103/1319-2442.344739 35532689

[47] Yun D, Park HS. Culture and the theory of planned behaviour: organ donation intentions in Americans and Koreans. J Pac Rim Psychol 2010;4:130–7. 10.1375/prp.4.2.130

[48] Park HS, Smith SW. Distinctiveness and influence of subjective norms, personal descriptive and injunctive norms, and societal descriptive and injunctive norms on behavioral intent: a case of two behaviors critical to organ donation. Hum Commun Res 2007;33:194–218. 10.1111/j.1468-2958.2007.00296.x

[49] Britt RK, Britt BC, Anderson J. Theoretical implications addressing rural college students’ organ donation behaviors. J Health Psychol 2017;22:650–60. 10.1177/1359105315611953 26585861

[50] Hyde MK, White KM. Are organ donation communication decisions reasoned or reactive? A test of the utility of an augmented theory of planned behaviour with the prototype/willingness model. Br J Health Psychol 2010;15:435–52. 10.1348/135910709X468232 19769797

[51] Newton JD, Newton FJ, Ewing MT et al. Conceptual overlap between moral norms and anticipated regret in the prediction of intention: implications for theory of planned behaviour research, psychol. Health 2013;28:495–513. 10.1080/08870446.2012.745936 23256489

[52] Cialdini RB, Reno RR, Kallgren CA. A focus theory of normative conduct: recycling the concept of norms to reduce littering in public places. J Pers Soc Psychol 1990;58:1015–26. 10.1037/0022-3514.58.6.1015

[53] Jacob Arriola KR, Perryman JP, Doldren MA et al. Understanding the role of clergy in African American organ and tissue donation decision-making. Ethn Health 2007;12:465–82. 10.1080/13557850701616896 PMC3489162 17978944

[54] Gorsuch RL, Ortberg J. Moral obligation and attitudes: their relation to behavioral intentions. J Pers Soc Psychol 1983;44:1025–8. 10.1037/0022-3514.44.5.1025

