# Supplementary material for: An application of the Theory of Planned Behavior within a living organ donation education intervention for American Muslim communities: a pre–post evaluation
Source: Health Educ Res. 2026 Oct 16;41(6):cyag030. doi: 10.1093/her/cyag030 (PMC13674604; doi:10.1093/her/cyag030)
Supplement: Supplementary file 1 [file appendix_-_survey_items_cyag030.docx]

**LIVING ORGAN DONATION BELIEFS**

Please note that the questions in this entire section are about *living donation*.

**Behavioral Beliefs**

Please indicate your level of agreement with the following statements

| *If I were to donate an organ:* | **Completely agree** | **Somewhat agree** | **Somewhat disagree** | **Completely disagree** |
| --- | --- | --- | --- | --- |
| 1. I would be helping to save the life of another person | 🞆 | 🞆 | 🞆 | 🞆 |
| 2. I would be violating the dignity of my body | 🞆 | 🞆 | 🞆 | 🞆 |
| 3. On the day of Judgment I will be held accountable for the sins committed by the recipient of my donated organs | 🞆 | 🞆 | 🞆 | 🞆 |
| 4. I would be performing a good deed | 🞆 | 🞆 | 🞆 | 🞆 |
| 5. I would be interfering with the destiny (*qadr*) of another person | 🞆 | 🞆 | 🞆 | 🞆 |
| 6. I would be giving something away that is not mine to give | 🞆 | 🞆 | 🞆 | 🞆 |
| *If I were to donate an organ:* | **Completely agree** | **Somewhat agree** | **Somewhat disagree** | **Completely disagree** |
| 7. I would be fulfilling a societal need | 🞆 | 🞆 | 🞆 | 🞆 |
| 8. I would be putting my own health at risk | 🞆 | 🞆 | 🞆 | 🞆 |
| 9. I would be committing a sin | 🞆 | 🞆 | 🞆 | 🞆 |

**Normative Beliefs**

As you are answering the following questions, please consider the following scenario:

“A community member at your mosque has a son named Ahmed who has kidney failure. Ahmed is in need of kidney transplantation. After Friday prayer a community leader asks attendees to sign up for screening to assess whether they are a potential match for Ahmed. After screening you find out that you are a match and could potentially donate your kidney.”

Please rate the likelihood that each of the following would approve of you donating to Ahmed. Note: circle “not applicable” only if this relationship does not exist for you.

|  | **Extremely likely** | **Somewhat likely** | **Somewhat unlikely** | **Extremely unlikely** | **Not applicable** |
| --- | --- | --- | --- | --- | --- |
| 10. Allah | 🞆 | 🞆 | 🞆 | 🞆 |  |
| 11. Islamic jurists | 🞆 | 🞆 | 🞆 | 🞆 |  |
| 12. People at my mosque | 🞆 | 🞆 | 🞆 | 🞆 |  |
| 13. My spouse | 🞆 | 🞆 | 🞆 | 🞆 | 🞆 |
| 14. My parent(s) | 🞆 | 🞆 | 🞆 | 🞆 | 🞆 |
| 15. My sibling(s) | 🞆 | 🞆 | 🞆 | 🞆 | 🞆 |
| 16. My children | 🞆 | 🞆 | 🞆 | 🞆 | 🞆 |
| 17. My friends | 🞆 | 🞆 | 🞆 | 🞆 |  |

Considering the above scenario, you are now trying to make a decision.

Please indicate how important the approval of each of the following is to your decision. Note: circle “not applicable” only if this relationship does not exist for you.

|  | **Very much** | **Somewhat** | **A little** | **Not at all** | **Not applicable** |
| --- | --- | --- | --- | --- | --- |
| 18. Allah | 🞆 | 🞆 | 🞆 | 🞆 |  |
| 19. Islamic jurists | 🞆 | 🞆 | 🞆 | 🞆 |  |
| 20. People at my mosque | 🞆 | 🞆 | 🞆 | 🞆 |  |
| 21. My spouse | 🞆 | 🞆 | 🞆 | 🞆 | 🞆 |
| 22. My parent(s) | 🞆 | 🞆 | 🞆 | 🞆 | 🞆 |
| 23. My sibling(s) | 🞆 | 🞆 | 🞆 | 🞆 | 🞆 |
| 24. My children | 🞆 | 🞆 | 🞆 | 🞆 | 🞆 |
| 25. My friends | 🞆 | 🞆 | 🞆 | 🞆 |  |

**Control Beliefs**

Please indicate your level of confidence with each of the following statements.

| *If I were to donate an organ:* | **Very confident** | **Confident** | **Not confident** | **Not confident at all** |
| --- | --- | --- | --- | --- |
| 26. It will not be sold illegally | 🞆 | 🞆 | 🞆 | 🞆 |
| 27. My religious modesty requirement will be met during the transplantation process | 🞆 | 🞆 | 🞆 | 🞆 |
| 28. My body would be treated respectfully during the surgery | 🞆 | 🞆 | 🞆 | 🞆 |
| 29. The organ will undergo procedures to make sure it is free of disease | 🞆 | 🞆 | 🞆 | 🞆 |
| 30. The chances of a successful transplantation to the recipient are high | 🞆 | 🞆 | 🞆 | 🞆 |

**VI. INTENTION**

Please answer the following question.

|  | **I have never thought about it** | **It has crossed my mind, but I do not know what to think** | **I have thought about it, but I would like to know more** | **I have thought about it frequently and I know the benefits and risks** | **I have thought about it a lot, and I am ready to make a decision if needed** |
| --- | --- | --- | --- | --- | --- |
| 31. Thinking about where you are today, which of the following best describes your thoughts on living organ donation? | 🞆 | 🞆 | 🞆 | 🞆 | 🞆 |

Please indicate how likely you are to do each of the following actions in the future.

|  | **Extremely likely** | **Somewhat likely** | **Somewhat unlikely** | **Extremely unlikely** |
| --- | --- | --- | --- | --- |
| 32. Donate your kidney | 🞆 | 🞆 | 🞆 | 🞆 |
| 33. Encourage a loved one with end-stage renal disease (ESRD) to seek out a donor | 🞆 | 🞆 | 🞆 | 🞆 |
| 34. Encourage a co-worker with end-stage renal disease (ESRD) to seek out a donor | 🞆 | 🞆 | 🞆 | 🞆 |
| 35. Encourage someone at your mosque with end-stage renal disease (ESRD) to seek out a donor | 🞆 | 🞆 | 🞆 | 🞆 |
